# The Synergistic Effect of Cyclic Tensile Force and Periodontal Ligament Cell-Laden Calcium Silicate/Gelatin Methacrylate Auxetic Hydrogel Scaffolds for Bone Regeneration

**DOI:** 10.3390/cells11132069

**Published:** 2022-06-29

**Authors:** Jian-Jr Lee, Hooi-Yee Ng, Yen-Hong Lin, Ting-Ju Lin, Chia-Tze Kao, Ming-You Shie

**Affiliations:** 1 School of Medicine, China Medical University, Taichung City 406040, Taiwan; d33977@mail.cmuh.org.tw; 2 Department of Plastic & Reconstruction Surgery, China Medical University Hospital, Taichung City 404332, Taiwan; 3 Department of Education, China Medical University Hospital, Taichung City 404332, Taiwan; hooiyeen@gmail.com; 4 The Ph.D. Program for Medical Engineering and Rehabilitation Science, China Medical University, Taichung City 406040, Taiwan; roger.lin0204@gmail.com; 5 Graduate Institute of Biomedical Sciences, China Medical University, Taichung City 406040, Taiwan; rubylin0423@gmail.com; 6 School of Dentistry, Chung Shan Medical University, Taichung City 40201, Taiwan; 7 Department of Stomatology, Chung Shan Medical University Hospital, Taichung 40201, Taiwan; 8 School of Dentistry, China Medical University, Taichung City 406040, Taiwan; 9 x-Dimension Center for Medical Research and Translation, China Medical University Hospital, Taichung City 404332, Taiwan; 10 Department of Bioinformatics and Medical Engineering, Asia University, Taichung City 41354, Taiwan

**Keywords:** cyclic tensile stimulation, YAP, calcium silicate, auxetic scaffold, bone regeneration

## Abstract

The development of 3D printing technologies has allowed us to fabricate complex novel scaffolds for bone regeneration. In this study, we reported the incorporation of different concentrations of calcium silicate (CS) powder into fish gelatin methacrylate (FGelMa) for the fabrication of CS/FGelMa auxetic bio-scaffolds using 3D printing technology. Our results showed that CS could be successfully incorporated into FGelMa without influencing the original structural components of FGelMa. Furthermore, it conveyed that CS modifications both the mechanical properties and degradation rates of the scaffolds were improved in accordance with the concentrations of CS upon modifications of CS. In addition, the presence of CS enhanced the adhesion and proliferation of human periodontal ligament cells (hPDLs) cultured in the scaffold. Further osteogenic evaluation also confirmed that CS was able to enhance the osteogenic capabilities via activation of downstream intracellular factors such as pFAK/FAK and pERK/ERK. More interestingly, it was noted that the application of extrinsic biomechanical stimulation to the auxetic scaffolds further enhanced the proliferation and differentiation of hPDLs cells and secretion of osteogenic-related markers when compared to CS/FGelMa hydrogels without tensile stimulation. This prompted us to explore the related mechanism behind this interesting phenomenon. Subsequent studies showed that biomechanical stimulation works via YAP, which is a biomechanical cue. Taken together, our results showed that novel auxetic scaffolds could be fabricated by combining different aspects of science and technology, in order to improve the future chances of clinical applications for bone regeneration.

## 1. Introduction 

Precision medicine is the current trend that can tailor the aspects of treatment and disease prevention according to one’s genetics, environment, and lifestyle [1]. There are various components of precision medicine, among which, stem cell-based therapies use stem cell-based products to develop personalized therapies for a unique individual [2,3]. From the concept, the stem of an individual differs between person-to-person and between populations, and therefore, the treatment should be modified and adjusted to effectively treat an individual uniquely. Stem cells exhibit unique pluripotency and self-regeneration ability, and therefore numerous studies have attempted to use the stem cells for the treatment of degenerative diseases with the hope of discovering novel treatment strategies for these diseases. Amongst the various types of stem cells, mesenchymal stem cells (MSCs) are commonly used due to their capability to differentiate into specific cell lineages under well-defined scenarios [4]. MSCs can be obtained from different sources such as the bone marrow, adipose tissues, or umbilical cord and can be induced into different cell types of the mesoderm such as bone, cartilage, and tendon. Due to its osteogenic capabilities, MSC is thus commonly applied for bone regenerative studies and clinical applications such as large bone defects [5].

Bone defects are commonly caused by infection, trauma, or iatrogenic procedures, and current treatment strategies for large bone defects include bone graft or bone replacement [6]. However, bone grafting is severely restricted by multiple limitations such as limited healthy sources and the requirement for multiple surgeries [7]. Therefore, there is a need for novel regeneration strategies for bone defects, and one of the potential treatment strategies is stem cell-based tissue engineering. Thus, scientists are currently attempting to manipulate the cells by controlling the local micro-environment and simultaneously understanding the interactions between the environment, factors, and cells [8]. A number of factors including biochemical cues, cell–extracellular matrix (ECM) contacts, cell–cell contacts, and the presence of mechanical forces have been proved to greatly influence stem cell differentiation [9]. Mechanical forces are involved in organogenesis during embryonic development and the existence of mechanical forces was shown to influence and regulate stem cell differentiation and proliferation [10]. Numerous studies have reported that the application of external mechanical forces to both matured and embryonic tissue models produced similar regenerative results, thus strongly indicating that the stem cells require the physical interactions of ECM to achieve effective differentiation and proliferation [11,12]. Thus, synthetic bio-models, imitating the different tissues have been developed in which the different mechanical stimuli, such as shear stress, compressive stress, and tensile stress could be applied to the tissue model. In addition to extrinsic forces, cells were also known to exert intrinsic forces onto ECM and neighboring cells through various mechanisms such as actomyosin contraction and ECM rearrangement. Therefore, it was suggested that further studies are required in order to further understand the relationship between mechanical stimuli and the fate of the stem cells [13]. 

It was understood that several key criteria should also be considered when fabricating the synthetic tissue models with extrinsic mechanical stimuli [14,15]. Thus, so far, it was understood that extrinsic mechanical stimuli would interact with the specific cell receptors known as mechanotransduction receptors and pathways. The mechanical forces are thus known as the easiest to manipulate and control as it is now able to modify the stiffness, degradation rate, and swelling capability of hydrogels by using appropriate hydrogels and modifications. Gelatin is a common natural biomaterial mainly used in soft tissue engineering due to its RGD motifs, excellent biodegradability, and bioactivity [16,17,18]. However, gelatin is known to possess weak mechanical properties, thus making it structurally unstable for clinical applications [19]. Therefore, scientists have attempted to modify gelatin with methacrylate to render the photo-polymerizable properties in order to improve the mechanical properties of gelatin. In our previous study, calcium silicate (CS) extracts were mixed with gelatin methacylol (GelMa), bioprinted, and then cross-linked using UV to form stable hydrogels for bone regeneration [20]. The physical and biological characteristics of the CS/FGelMa hydrogels were thoroughly investigated and reported in our previous report. It was suggested that CS/FGelMa hydrogels have enhanced biological and mechanical properties and were known to be superior in bone regeneration capabilities when compared to CS or GelMa hydrogels alone. In addition, CS/FGelMa had the capability to release calcium and silicate ions into its surrounding fluids, which was shown to promote the osteogenesis and angiogenesis capabilities of scaffolds.

Generally, bone tissue consists of a network of osteocytes, osteoblasts, and osteoclasts. Osteocytes serve as sensory cells responsible for mechanotransduction, whereas the other type of cells function as effector cells for bone remodeling. Mechanical loading creates hydrostatic pressure through the lacunae canals which are then sensed by osteocytes. In response, osteocytes secrete paracrine signaling molecules which would modulate the activity of osteoclasts and osteoblasts. In biological terms, these pulsatile forces would activate the osteocytes via activation of the membrane receptor, which is functioned by stretch-activated ion channels coupled with g-protein and integrin-coupled cytoskeleton. Downstream mitogen-activated protein kinase (MAPK) pathways including ERK1/2, p38, and c-Jun amino (N)-terminal kinases (JNK) would be then recruited and activated to upregulate the proliferation and differentiation of mechanoresponsive cells. Previous studies revealed that MSCs were mechanosensitive and that extrinsic mechanical stimuli were able to influence the cellular morphology, phenotype, and downstream cellular function [21]. Further in vitro studies showed that MSCs exhibited the enhanced expression and secretion of osteogenic-related genes and proteins when extrinsic mechanical stimuli were applied, thus enhancing the capability of bone regeneration [22]. According to our knowledge, so far there have been no more studies combining the tensile biomaterials, stem cells, and extrinsic mechanical stimulation for bone regeneration. 

**Figure 1:**
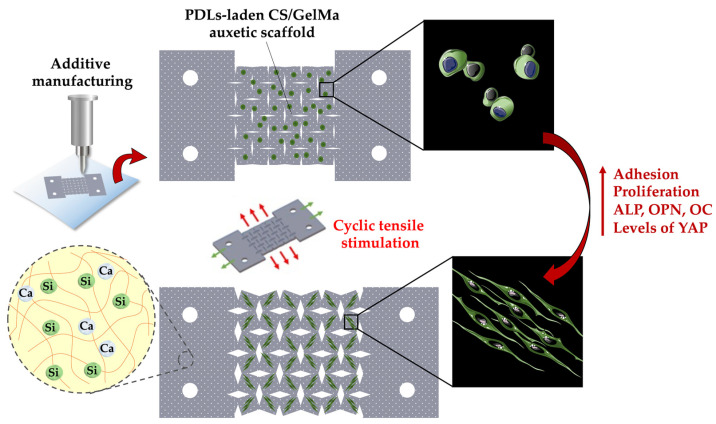
Schematic diagram of the 3D printed CS/FGelMa auxetic scaffold using the proposed structural design, which endowed the scaffolds with the ability to activate the YAP protein by the cyclic tensile stimulation and also enhanced osteogenic-related protein expression.

In this study, we incorporated mechanical stimulation into our CS/FGelMa hydrogels and evaluated its potentiality in enhancing bone regeneration. This study was a continuation of our previous work and the aim of this study was to apply and understand the effects of extrinsic mechanical stimuli on bone regeneration, in order to bring bone regeneration to the next level [23]. In the first part of this study, we evaluated the composition, mechanical properties, and degradation rates of CS/FGelMa hydrogels. After this, human periodontal ligament cells (hPDLs) were encapsulated into the hydrogel and fabricated into auxetic scaffolds. hPDLs are MSCs derived from the periodontal ligament, and they are reported to possess similar osteo, chondro, and adipo-inductive capabilities as MSCs. In addition, hPDLs had been clearly demonstrated to mediate various cellular responses to mechanical force stimulation [24]. However, the hPDLs express several osteogenic-related phenotypic biomarkers in vitro and suggest that mechanical force stimulation alone can influence the differentiation of hPDLs to osteoblast cells [25]. Then, extrinsic mechanical stimulation was applied to the auxetic scaffolds and osteogenic markers were evaluated to determine its capability for bone tissue engineering (Figure 1). Based on the results obtained from this study, it was reported that the novel hydrogels could be fabricated by combining the different factors and biomaterials to bring the tissue regeneration to the next level.

## 2. Materials and Methods

### 2.1. Synthesis of Photo-Polymerizable FGelMa

The FGelMa used in this study was fabricated according to the protocols reported in our previous study [26]. Firstly, fish gelatin (Sigma-Aldrich, CAT#SLCJ6149, St. Louis, MO, USA) was dissolved in 250 mL of cold distilled water at a concentration of 15 wt% and stirred at 50 °C for 2 h at 100 rpm. A ratio of 0.6 g of methacrylic anhydride (Sigma-Aldrich, Lot#STBK0716, St. Louis, MO, USA): 1 g of gelatin was then added to the fish gelatin solution and stirred at 50 °C for 2 h at 100 rpm in a dark environment. Then, 250 mL of distilled water was added and the FGelMa solution was centrifuged to remove the unreacted methacrylic anhydride. After this, the FGelMa was dialyzed at 40 °C in deionized water for 3 days in order to further remove the unreacted methacrylic anhydride. The pH of the FGelMa solution was adjusted to 7.4, lyophilized, and stored at −20 °C in a refrigerator until further usage.

### 2.2. Synthesis of Calcium Silicate (CS) Powder

CS powder is normally composed of calcium oxide (CaO, CAT#422830025, Thermo Scientific, Waltham, MA, USA), silicon dioxide (SiO_2_, Lot#4856-050117, NanoAmor, Houston, TX, USA), and aluminum oxide (Al_2_O_3_, Lot#0000265147, Panreac Química, Barcelona, Spain). The above compounds were evenly mixed and stirred, placed in a sintering furnace, and sintered at 1400 °C for 2 h. The sintered compound was then cooled for 1 h, mixed with anhydrous alcohol, centrifuged, and ground using a planetary ball mill for 8 h to obtain CS powder. The CS powder was then stored in a dry environment until further usage. 

### 2.3. Preparation of CS/FGelMa Bioink

Photo-initiator lithium phenyl-2,4,6-trimethylbenzoylphosphinate (LAP, Sigma-Aldrich, Lot#000026755, St. Louis, MO, USA) was mixed with distilled water at a concentration of 0.25% *w*/*w* and stirred in 50 °C until the complete dissolution. Then, FGelMa at a concentration of 10% *w*/*w* was added and stirred until the complete dissolution. The above procedures were conducted in a dark environment and 0, 0.5, and 1% *w*/*v* of CS powder was added. The obtained samples in this study were then referred as CS0, CS0.5, and CS1 according to the concentrations of CS.

### 2.4. Characterization of CS/FGelMa Scaffold

The ionic and molecular groups of CS/FGelMa were evaluated using a ^1^H NMR (nuclear magnetic resonance) spectrometer. The signal intensities of methacryloyl groups and lysine with chemical shifts of 5–6 and 2.8–3.1 ppm were evaluated. In addition, Fourier transform infrared spectroscopy (FTIR, Vertex 80v, Bruker, Germany) was used to analyze the chemical structure and functional groups of the samples in the wavelength range of 4000~500 cm^−1^. Furthermore, XRD (Bruker D8 SSS, Karlsruhe, Germany) was used to perform the diffraction analysis of the crystalline phase of CS/FGelMa to determine its atomic and molecular structure. In addition, the CS/FGelMa was printed into dumbbell-shaped specimens and stretched from both ends using a dynamic mechanical analyzer DMA Q800 (TA Instruments, Newcastle, DE, USA) at a fixed rate of 1 mm/min. The specimens were stretched till they tore in the middle, and the stress–strain graph was then plotted using the data. After this, the tensile strength and Young’s modulus were then calculated by the software. Six specimens from each group were tested and the mean and standard deviation were recorded and evaluated.

### 2.5. In Vitro Degradation Behaviour of CS/FGelMa Scaffold

The in vitro degradation behavior of each group was tested at 0, 3, 7, and 14 days of immersion. The hydrogels were rinsed with deionized water, lyophilized, and weighed to obtain the dry weight W0. The hydrogels were placed into 20 mL of Dulbecco’s modified minimal essential medium (DMEM, Gibco, Carlsbad, CA, USA) in a 50 mL centrifuge tube and placed into a water bath at 37 °C. At 0, 3, 7, and 14 days of immersion, the hydrogels were removed, lyophilized, and weighed to obtain the dry weight Wd. The degradation rates were then calculated using the following formula:

Weight loss (%) = (W0 − Wd)/W0 × 100%

Eight specimens from each group were tested and the mean and standard deviation were recorded and evaluated.

### 2.6. Cell Culture

Primary human periodontal ligament cells (hPDLs; ScienCell Research Laboratories, Carlsbad, CA, USA) were used in this study and cultured in the recommended commercial medium (#2301; ScienCell Research Laboratories, Carlsbad, CA, USA). Cells used in this study were from the third to eighth subculture generation. For subsequent studies, 5×10^6^ per mL of cells were encapsulated homogeneously into CS/FGelMa bioink.

### 2.7. Fabrication of hPDLs-Laden CS/FGelMa Auxetic Scaffolds and Cyclic Tensile Stimulation

F127 (30 wt%, Sigma-Aldrich, St. Louis, MO, USA) was used to form a supportive mold for the auxetic scaffold. The hPDLs-laden CS/FGelMa was cast into the mold and UV (SP-11, USHIO, Japan) at a wavelength of 320–500 nm, 45% output, and 1.77 Watt was used to photo-polymerize the GelMA scaffold for 90 s. After which, the F127 was dissolved by placing the entire construct into cold sterile deionized water. The fabricated auxetic scaffold was then mounted onto the dynamic culture system (ATMS Boxer QQA Cyclic Stretch Culture System, Genemessenger, Kaohsiung, Taiwan) and exposed to 0.5 Hz frequency and 10% deformation to simulate extrinsic periodic mechanical stimulation. The medium was changed every 2 days during the study period.

### 2.8. Cell Viability and Morphology

The auxetic scaffold was removed from the dynamic culture system and rinsed twice with phosphate buffer solution (PBS, Gibco, Carlsbad, CA, USA). Cell viability was quantified using a PrestoBlue assay (Invitrogen, Carlsbad, CA, USA). In brief, 500 μL of the PrestoBlue reagent and DMEM at a ratio of 1:9 was added to the auxetic scaffold and placed in a 5% CO_2_ incubator at 37 °C for 1.5 h. Then, 100 µL was aspirated into a new 96 well plate and the absorbance was measured using a spectrophotometer at 570 nm wavelength. 

In addition, via cell morphology of hPDLs in auxetic scaffolds, the specimens were fixed with 4% paraformaldehyde and then washed with cold PBS. The scaffolds were permeabilized by incubation with 0.1% Trition X-100 in PBS for 15 min. The cytoskeleton and nuclei of cells were stained with phalloidin (Alexa Fluor 488 phalloidin, Invitrogen, Carlsbad, CA, USA). In addition, 300 nM of 4′,6-diamidino-2-phenylindole (DAPI, Invitrogen, Carlsbad, CA, USA) was used to stain the cell nuclei. These dyes were made according to the manufacturer’s instructions, and the morphology of hPDLs was visualized by using a confocal microscope (Leica TCS SP8, Wetzlar, Germany).

### 2.9. Immunofluorescence Staining

In this experiment, alpha-smooth muscle actin (α-SMA), phalloidin, and DAPI were stained to observe the proliferative capability of hPDLs cultured in the auxetic scaffolds. In brief, the medium was aspirated and replaced with 4% paraformaldehyde for 30 min to allow cell fixation. Then the solution was replaced with 0.1% Triton for 15 min to cause cell lysis and subsequently replaced with primary anti-α-SMA (1:200, ab5694, Abcam, Cambridge, MA, USA) followed by anti-rabbit conjugated tetramethylrhodamine (1:1000, TRITC, Invitrogen, Carlsbad, CA, USA), phalloidin, and DAPI. The above study was conducted in a dark environment and photo images of the immunofluorescence staining were taken using a conjugate focusing microscope (Leica TCS SP8 X, Wetzlar, Germany).

### 2.10. Western Blot

The auxetic scaffold was removed from the dynamic culture system and rinsed twice with PBS after being cultured for 1 day. Then, 100 µL of radioimmunoprecipitation assay buffer (RIPA buffer, Gibco, Carlsbad, CA, USA) was added for 5 min on an ice bath to cause cell lysis. After this, the scaffold was centrifuged at 13,000 rpm for 15 min at 4 °C and the supernatant was subsequently removed, leaving the protein pellet. Then, BCA protein quantification was done to obtain the concentration of the various proteins. In brief, 5 µL of the protein sample was added to a fresh 96-well plate, and 200 µL of the assay reagent (Protein assay reagent A + Reagent B) was added and left to react in an incubator at 37 °C for 30 min. After this, the absorbance was measured using a spectrophotometer at 570 nm wavelength. In addition, 30 µg of protein sample was added to the sample buffer, heated at 95 °C for 5 min, aspirated into 10% SDS-PAGE, and electrophoresis was done at a voltage of 80 volts with a BIORAD system for 2 h. The proteins were then transferred onto polyvinylidene fluoride membrane (PVDF) at 100 volts for 1.5 h. The membrane was then blocked with 5% BSA at room temperature for an hour. The primary antibodies anti-p-FAK (1:1000, 44-624G, Invitrogen, Carlsbad, CA, USA), anti-FAK (1:1000, AHO0502, Invitrogen, Carlsbad, CA, USA), anti-pERK1/2 (1:1500, 13-6200, Invitrogen, Carlsbad, CA, USA), anti-ERK (1:1500, 44-680G, Invitrogen, Carlsbad, CA, USA), and anti-β-actin (1:3000, MA5-11869, Invitrogen, Carlsbad, CA, USA) diluted in TBST were then added and then left to react overnight at 4 °C. After this, it was rinsed and images of the membrane were taken using a camera.

### 2.11. Osteogenic Markers 

The hPDLs-laden auxetic scaffolds were cultured in an osteogenic medium in the dynamic culture system. The capability of osteogenic differentiation was evaluated using an osteogenesis assay kit (StemPro™ osteogenesis differentiation kit, Invitrogen, Carlsbad, CA, USA). In brief, 0.2% NP40 was added to cause cell lysis, and centrifuged at 6000 rpm for 15 min. Then, 1 M diethanolamine buffer was mixed in each sample, and 3M NaOH was added after 30 min to stop the reaction. The absorbance was quantified using a spectrophotometer at 405 nm. In addition, secretion of osteopontin (OPN, San Diego, CA, USA) and osteocalcin (OC) (MyBioSource, San Diego, CA, USA) from hPDLs at different time points was determined using an enzyme-linked immunosorbent assay according to the manufacturer’s instructions.

### 2.12. Inhibition of YAP 

In the earlier results, we considered that the presence of tensile stimulation of hPDLs was through the activation of hPDL’s YAP protein, a mechanosensitive transcriptional activator with a critical role in the cell behaviors. Thus, in the end, we exposed the hPDLs-laden CS/FGelMa auxetic scaffold to a YAP inhibitor, Veteprofin (MedChemExpress, Monmouth Junction, NJ, USA). After culturing for 3 days, we used immunofluorescence staining and osteogenic-related proteins to assess the role of YAP in tensile stimulation.

### 2.13. Statistical Analyses

A one-way statistical analysis of variance (ANOVA) was applied to analyze the significance of the differences between the groups in each experiment. Determination of the significant deviations of each sample was made using Scheffe’s multiple comparison test. The statistical solutions showed that a *p*-value < 0.05 could be statistically considered as significant, as indicated by an *.

## 3. Results and Discussion

### 3.1. Characterizations of CS/FGelMa Scaffold

**Figure 2:**
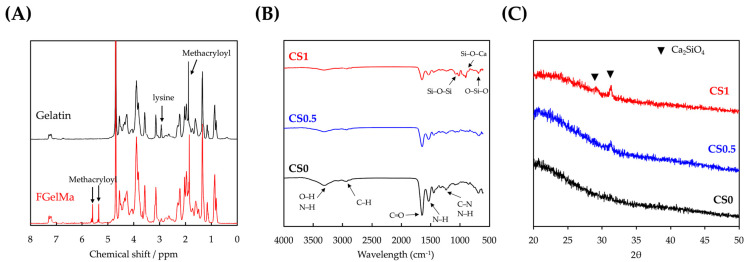
(**A**) The ^1^H NMR spectra of gelatin and FGelMa; (**B**) FTIR; and (**C**) XRD for CS0, CS0.5, and CS1.

In this study, we attempted to fabricate CS/FGelMa and evaluate the effects of biomechanical stimulation on bone tissue engineering. The formation of the sample was confirmed by using various techniques. The ^1^H NMR spectra of fish gelatin and FGelMa as shown in Figure 2A confirmed the successful formation of the modified FGelMa. In comparison with the ^1^H NMR spectra of gelatin, there were new proton peaks corresponding to methacryloyl groups around 5–6 and at 1.9 ppm in fish gelatin. In addition, it was observed that the free lysine signal of FGelMa in 2.8–3.1 ppm range decreased markedly. These results showed that the methacryloyl groups were successfully functionalized onto gelatin [27]. GelMa has been investigated as a potential alternative for tissue engineering and thus numerous studies have been reported emphasizing the versatility of GelMa for tissue engineering, drug delivery, and 3D printing applications. Most importantly, FGelMa showed significant features such as biocompatibility, enzymatic degradation in response to matrix metalloproteinases, availability of RGD sequences for cellular adhesion, and tailorable mechanical properties. However, usage of gelatin in bone tissue engineering was limited due to its instability and poor mechanical properties when compared to the mechanical properties of native bones. Hence, in this study, we attempted to modify FGelMa to further improve its bone tissue regenerative capabilities. 

The FTIR and XRD results for the various CS loaded FGelMa (CS0, CS0.5 and CS1) are shown in Figure 2B,C, respectively. Similarly, these analyses were also done to confirm the successful loading of CS into FGelMa and investigated the interactions between CS and FGelMa. On comparison with CS0, there were new Si-O-Si, Si-O-Ca, and O-Si-O bonds at 1200, 950, and 750 cm^−1^ in both CS0.5 and CS1 groups [20]. In addition, with respect to the intensity of the peaks in the samples corresponding to the concentrations of CS it was explained that both CS0.5 and CS1 had weak imide bonds of FGelMa when compared to CS0. Obvious Ca_2_SiO_4_ diffraction peaks were observed at 29.4° and 30.5° in CS1. On the other hand, there were no such peaks observed in the CS0 group [23]. These results were considered significantly important as they showed that CS was successfully loaded onto FGelMa and such a modification did not alter the initial structural characteristics of FGelMa. Furthermore, the bonds between CS and FGelMa were noted to be generally covalent and ionic bonds. Based on this, it was hypothesized that the CS modifications carried out in this study were able to enhance both the biological and mechanical properties of FGelMa. It was known that CS is another common inorganic biomaterial used in bone engineering. According to our knowledge, there were only a very few reports combining CS with FGelMa and thus the aim of this study was to combine an inorganic material with an organic biomaterial for bone tissue engineering. For consideration, it was suggested that both the materials should have characteristics that would complement each other to improve the regenerative capabilities of the biomaterials.

### 3.2. Mechanical and Degradation Properties of CS/FGelMa Scaffold

**Figure 3:**
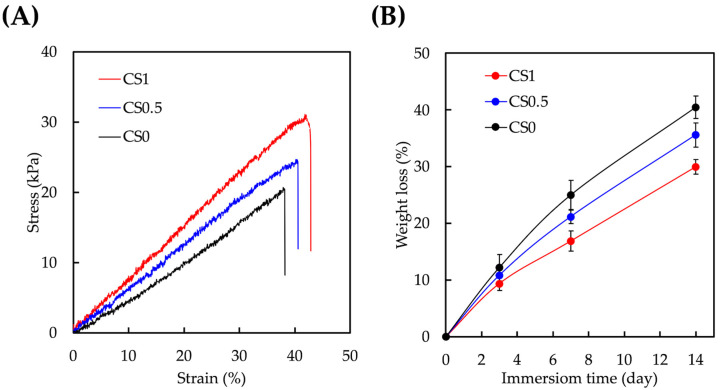
(**A**) Tensile stress–strain curves and (**B**) weight loss profiles of CS0, CS0.5, and CS1. Data presented as mean ± SEM, *n* = 6 for each group.

An elastic modulus test was performed on dumbbell-shaped specimens of the various groups and the obtained stress–strain curves are as seen in Figure 3A. As expected, there was an increase in mechanical properties according to the concentrations of CS added. It was found that CS0 had a mechanical strength/elastic modulus of 20.7 ± 1.9/47.9 ± 3.5 kPa whilst CS0.5 and CS1 had a mechanical strength/elastic modulus of 24.8 ± 1.6/64.0 ± 4.6 kPa and 31.2 ± 2.1/75.1 ± 5.3 kPa, respectively. In addition, CS0 had a typical brittle stress–strain curve which was indicated by a steep slope followed by a rapid decline and thus indicating that the specimen was unable to support the increase in load. It was hypothesized that the presence of covalent bonds from CS could enhance the mechanical properties of FGelMa [28]. Bone tissue is such an intricate tissue that a simple hydrogel without any modifications would not be able to meet the requirements for bone regeneration. Gaharwar et al. first started designing an enhanced inorganic nanoparticle FGelMa via photo-crosslinking and the results showed that such a modification significantly improved the mechanical stiffness of FGelMa by 10 folds and mechanical toughness by 20 folds [29]. It was reported that the presence of imide bonds and carboxylate-amine interactions could contribute to the increase in the mechanical properties and subsequently lead to the increased alkaline phosphatase activity and mineralization. However, more studies still need to be done in order to make the hydrogels suitable for bone tissue engineering. The enhanced mechanical strength of CS/FGelMa composite hydrogels made it more appropriate for clinical applications and also for better surgical handling during the implantations. 

FGelMa was chosen in this study as the hydrogel could allow for cell encapsulation, which was something not achievable using CS alone [30]. The degradation rate of the various hydrogels is shown in Figure 3B. The residual weights of CS0, CS0.5, and CS1 after 14 days of immersion were 59.6 ± 1.9%, 64.4 ± 2.1%, and 70.1 ± 1.3% respectively. It was stated that both the presence of CS and increasing the content of CS could prolong the degradation rate of the hydrogels. Similarly, it was hypothesized that the presence of covalent bonds from CS contributed significantly to the stability of the hydrogels. In addition, the above results showed that the ratio of CS to FGelMa could be modified to alter the degradation rates to better suit the needs of different applications. Scaffold degradation is a key component of bone tissue engineering [31]. The implanted scaffolds were temporary, and the degradation rate of the scaffolds should match the tissue regeneration rates and also yet to provide adequate mechanical support during the regeneration. Depending on the types of fracture and wounds, the average time required for complete bone regeneration would be approximately 3 to 8 weeks. These results showed that the addition of CS successfully improved the mechanical properties and degradation rates of scaffolds by making them more suitable for bone tissue engineering.

### 3.3. Cell Proliferation and Morphology

**Figure 4:**
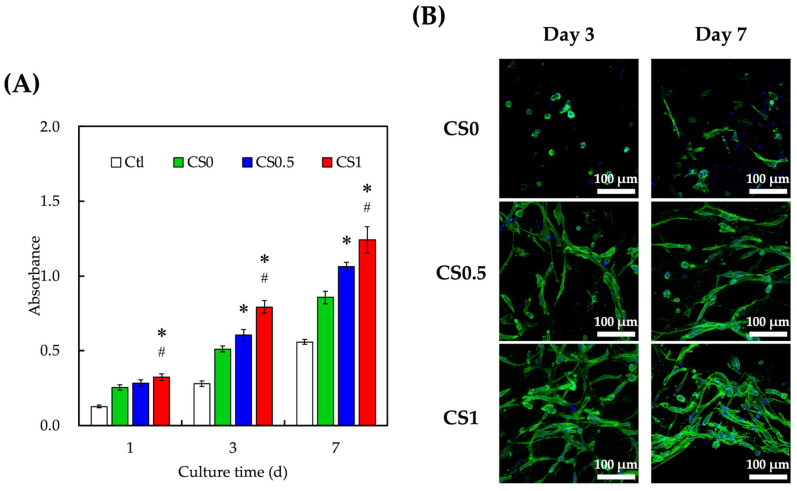
(**A**) Proliferation rates and (**B**) cell morphology of hPDLs-laden auxetic scaffolds. hPDLs growing on traditional culture dishes were used as Ctl. Data presented as mean ± SEM, *n* = 6 for each group. A *p*-value of <0.05 was considered as a significant difference according to the calculation of Scheffe’s multiple comparison test. * represents significant differences when compared to CS0 and # represents significant differences when compared to CS0.5. Immunofluorescence images showed localization of F-actin and nucleus on day 3 and day 7 (green: F-actin; blue: nucleus; scale bar is 100 µm).

In order to evaluate the cellular proliferation, hPDLs were encapsulated in the auxetic scaffold and cultured at different time points. The proliferation and live/dead staining are shown in Figure 4. After 1 day of culture, the cellular proliferation of hPDLs cells in CS1 was significantly increased to more than 10% higher proliferation when compared to CS0.5 and CS0. This phenomenon was also observed on days 3 and 7 of culture. CS0.5 was observed to have significantly higher proliferation from day 3 of culture onwards. After 7 days of culture, CS1 was found to have 45% and 16% higher proliferation when compared to CS0 and CS0.5, respectively. In addition, it was noted that the hPDLs were generally well adhered to in the FGelMa after 3 days of culture. Cells were in a long spindle shape when compared to the clustered round shape of hPDLs in CS0. Furthermore, it was noted that there was more cell nucleus in both CS0.5 and CS1 on both days 3 and 7 when compared to CS0, further confirming our quantification results discussed above. CS-based biomaterials were widely used in bone regeneration as it has been shown to promote the adhesion, proliferation, and differentiation of human mesenchymal stem cells and osteoblasts-related cells [32,33,34]. It was previously shown in our prior publications that CS were able to release Ca and Si ions into its surrounding fluids, which would act as key regulators for cellular responses to CS-based biomaterials. Both Ca and Si ions were potent regulators of cellular activities such as proliferation and differentiation of stem cells. In addition, Si ions were known to play a huge role in the early stages of bone formation and calcification [18,35]. According to our previous reports, CS-based biomaterials were shown to release both Ca and Si ions into their surrounding fluids, thus bringing about enhanced cellular activities.

### 3.4. Biomarker of Adhesion and Proliferation-Related Proteins

**Figure 5:**
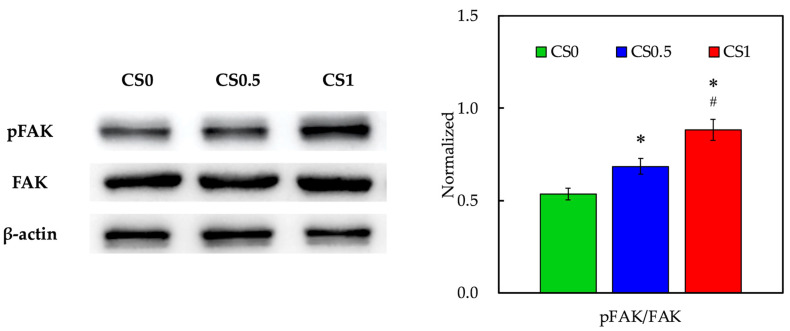
Western blot and quantification of pFAK and FAK expressions of hPDLs cultured in CS0, CS0.5, and CS1. Data presented as mean ± SEM, *n* = 6 for each group. Quantification of Western blot bands expressed as pFAK/FAK ratios. A *p*-value of <0.05 was considered as a significant difference according to the calculation of Scheffe’s multiple comparison test. * represents significant differences when compared to CS0 and # represents significant differences when compared to CS0.5.

The levels of FAK were evaluated using Western blot and the results are shown in Figure 5. As seen from the Western blot results, FAK bands were slightly enhanced in CS1 when compared to the others. However, pFAK bands were obviously enhanced in CS1 and CS0.5 when compared to CS0. The data after normalization showed that pFAK expressions were significantly increased in CS1 and CS0.5 when compared to CS0. Interestingly, pFAK expressions in CS1 were significantly increased when compared to CS0.5, by strongly indicating that the CS concentrations played a role in enhancing the cellular activities [36]. Phosphorylation of FAK was known to affect the downstream cellular behaviors such as migration, differentiation, and proliferation. In addition, FAK was reported to be involved in osteogenesis via multiple pathways. Kim et al. reported that FAK deficiency in osteoblasts-related cells led to delayed bone regeneration and remodeling [37]. Our previous results showed that the release of Si ions from CS was via activation of FAK and its downstream signaling molecules [38]. Therefore, these results showed that our CS/FGelMa scaffolds were able to activate similar mechanisms as their individual counterparts in order to enhance bone regeneration.

**Figure 6:**
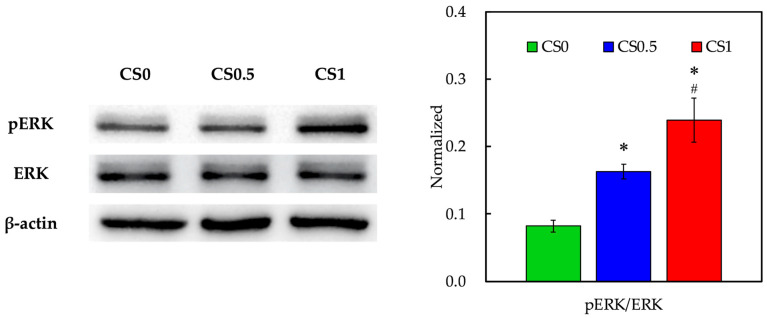
Western blot and quantification of pERK and ERK expressions of hPDLs cultured in CS0, CS0.5, and CS1. Data presented as mean ± SEM, *n* = 6 for each group. Quantification of Western blot bands expressed as pERK/ERK ratios. A *p*-value of <0.05 was considered as a significant difference according to the calculation of Scheffe’s multiple comparison test. * represents significant differences when compared to CS0 and # represents significant differences when compared to CS0.5.

In order to further investigate the downstream FAK-mediated pathway involved in the promotion of osteogenesis by biomechanical stimulation, ERK, and its phosphorylated counterpart expressions were evaluated as shown in Figure 6. As shown above, the addition of CS into FGelMa led to the activation of FAK via phosphorylation after culture. With the addition of biomechanical stimulation, CS1 showed higher expressions of pERK when compared to CS0.5 and CS0. Quantification results confirmed that CS1 showed significantly higher expressions of pERK when compared to both CS0.5 and CS0. On the other hand, CS0.5 showed significantly higher expressions of pERK than that of CS0. As reported by Chandran et al., FAK could act as an upstream signaling molecule for downstream ERK and Runx2 gene activation, in which activation of this pathway led to collagen synthesis and osteogenesis [39]. In addition, ERK was reported to be a central hub for regulating bone homeostasis and promoting the survival and differentiation of osteoblast by controlling the osteogenesis transcription factors. Another study reported by Chen et al. indicated that ERK and p38 were activated by external stimuli such as extracorporeal shock waves, which could subsequently activate the downstream osteogenic factors and mechanical stimulated proliferation and differentiation of bone-related cells [40]. Further in vivo studies showed that extracorporeal shock waves could promote the healing of segmental fractures in rats by promoting bone morphogenetic proteins. In this study, it was shown that the addition of CS into FGelMa could enhance the FAK activation whilst application of external biomechanical stimulation further promoted the downstream ERK activation. Taken together, it was proposed that the CS/FGelMa scaffolds with biomechanical stimulation reported in this study possessed the potential to enhance osteogenesis via the above-mentioned pathways.

### 3.5. Effect of Cyclic Tensile Stimulation on Cell Proliferation and Morphology

**Figure 7:**
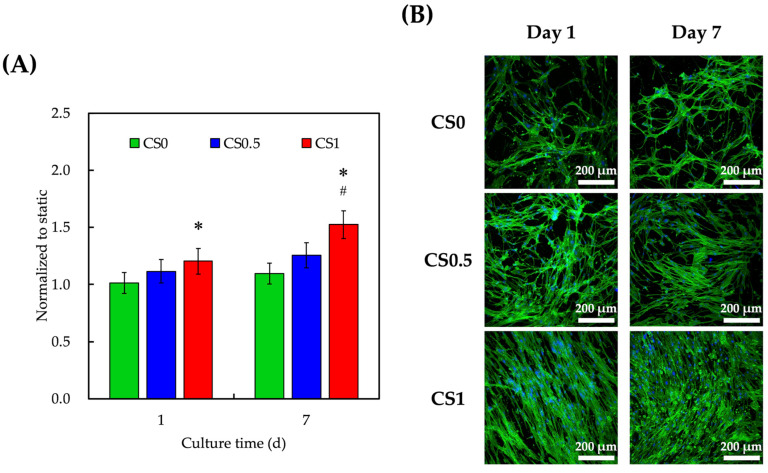
(**A**) Proliferation and (**B**) cell morphology of hPDLs-laden CS0, CS0.5, and CS1 after exposure to cyclic tensile stimulation. hPDLs growing on traditional culture dishes were used as Ctl. Data presented as mean ± SEM, *n* = 6 for each group. A *p*-value of <0.05 was considered as a significant difference according to the calculation of Scheffe’s multiple comparison test. * represents significant differences when compared to CS0 and # represents significant differences when compared to CS0.5. Immunofluorescence images showed localization of F-actin and nucleus on day 1 and day 7 (green: F-actin; blue: nucleus; scale bar is 200 µm).

The effect of tensile force load on hPDLs-laden auxetic scaffold was analyzed and the proliferation and live/dead staining are shown in Figure 7. Cells within the body were constantly exposed to various types of biomechanical stimuli and the stimuli might be tissue-specific and might vary according to different scenarios [41]. Therefore, scientists have attempted to better simulate the native micro-environment by externally applying the biomechanical stimulation to tissue culture. These stimuli were reported to stimulate and regulate cellular behaviors and activities [42]. In this study, we attempted to apply an external biomechanical stimulus to the CS/FGelMa hydrogels and evaluate its effect on bone tissue regeneration. It was found that after 1 day of culture, the cellular proliferation of hPDLs in CS1 was significantly higher (5%) when compared to CS0. Interestingly, after 7 days of culture, the cellular proliferation in CS1 was significantly increased when compared to both CS0.5 and CS0. The cell morphology showed that hPDLs in all groups were well-adhered in the hydrogel and the structure appeared as long spindle shapes. In the static culture, cells in CS0 were noted to be round and clustered in groups which indicated poor adhesion. It was conveyed that biomechanical stimulation alone was able to improve the cellular adhesion for CS0. After 7 days of culture, all groups obviously had the increased number of cells and long flat mitotic spindles. Thorpe et al. reported that the different types of cellular responses could be elicited by varying the types of stimuli [43]. In their study, the dynamic compressions were applied to hydrogels and thereby influencing the stem cells to have chondrogenic differentiation instead of the usual myogenic expressions [44]. Therefore, it was suggested that even though biomechanical stimuli might be advantageous in many situations, the cellular responses would be mainly dependent on duration, magnitude, and frequency of the stimuli.

### 3.6. Effect of Cyclic Tensile Stimulation on α-SMA Expression

**Figure 8:**
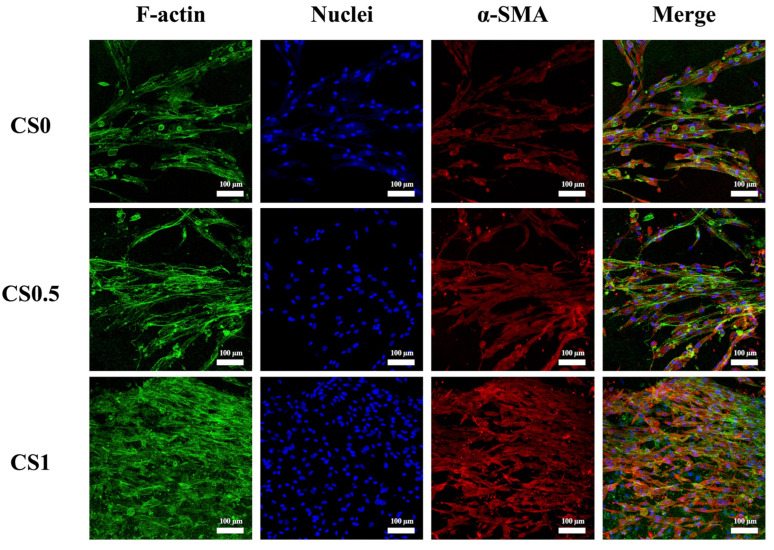
Immunofluorescence staining of α-SMA expression of hPDLs-laden various CS-contained FgelMa auxetic scaffolds with cyclic tensile stimulation for 3 days. Immunofluorescence images showed localization of F-actin, nucleus, and α-SMA on day 3 (green: F-actin; blue: nucleus; red: α-SMA; scale bar is 100 µm).

In fact, hPDLs have the capability to regenerate to multi-lineage cell differentiation. In order to evaluate the influence of tensile stimulation on hPDLs differentiation, the cells were stained with α-SMA and F-actin as shown in Figure 8. As seen, CS1 possessed the highest amount of α-SMA staining when compared to CS0.5 and CS0. In addition, F-actin cytoskeleton staining revealed that CS had the best adhesion as seen from its F-actin spreading and cytoskeleton development. Thus, it was important to note that hPDLs were highly multipotent stem cells as they had the capability to differentiate into osteoblasts, adipocytes, chondrocytes, fibroblasts, endothelial cells, etc. Huang et al. published a review article on the influences of biomechanical stimulation on hPDLs differentiation [45]. According to their study, for various durations, 0.1 to 0.5% of tensile forces were able to increase osteogenic, cardiomyogenic, and keratocyte markers whilst the compressive and vibration forces were able to enhance the collagen secretion and osteogenesis. It can be stated that during chewing or grinding of teeth, the mechanical stimulation is distributed and traveled through the teeth and alveolar bone to hPDLs [46]. Such mechanical forces were known to maintain the phenotypic and structural integrity of hPDLs. In addition, hPDLs were found to express α-SMA during wound healing and stress [47,48]. α-SMA cells were known to be mechanically active and reported to be involved in extracellular matrix remodeling [49]. α-SMA cells were also involved in early stages of tooth development where they were found to migrate to the alveolar bone crypt after the bell stage of tooth development and differentiate into osteoblasts. Therefore, it was demonstrated that the biomechanical stimulation was beneficial for enhancing the osteogenesis and tooth regeneration via increasing α-SMA expressions in hPDLs. 

### 3.7. Effect of Cyclic Tensile Stimulation on Osteogenesis

**Figure 9:**
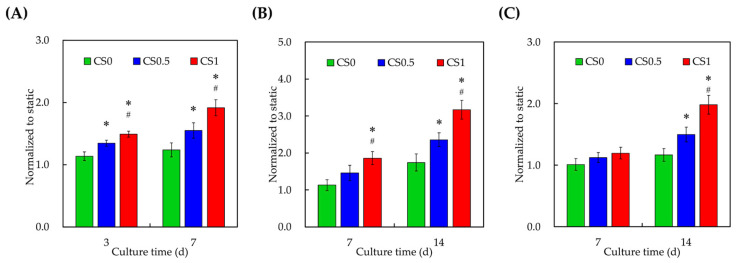
Levels of (**A**) ALP; (**B**) OPN; and (**C**) OC expressions from hPDLs cultured in CS0, CS0.5, and CS1 for 3 and 7 days. hPDLs growing on traditional culture dishes were used as Ctl. Data presented as mean ± SEM, *n* = 6 for each group. A *p*-value of <0.05 was considered as a significant difference according to the calculation of Scheffe’s multiple comparison test. * represents significant differences when compared to CS0 and # represents significant differences when compared to CS0.5.

The levels of osteogenic-related markers alkaline phosphatase (ALP), osteopontin (OPN), and osteocalcin (OC) were evaluated as shown in Figure 9. After 3 days of culture, CS1 was seen to have significantly higher levels of ALP and OPN when compared to CS0.5 and CS0 (Figure 9A,B). After 7 days of culture, CS1 had 1.6 and 1.3 times higher levels of ALP when compared to CS0 and CS0.5, respectively. For OPN (Figure 9B), CS1 was 2 and 1.5 times higher after 7 days of culture when compared to CS0 and CS0.5, respectively. ALP is an early osteogenic marker that is being involved in bone formation by degrading an osteogenic inhibitor inorganic pyrophosphate (PP(i)). This PP(i) is a potent inhibitor of hydroxyapatite formation by not contributing to inorganic phosphate which is also a critical molecule for osteoblast differentiation and mineralization. Therefore, it was understood that the increased levels of ALP meant higher levels of early hydroxyapatite formation and osteogenesis. OPN is also an early osteogenic marker with RGD containing adhesive glycoproteins. Recent studies reported that OPN was not only unique to bones, but it could also be found in dentin, cartilage, kidney, blood vessels, etc. It usually works by binding to ανβ3 integrins via their RGD glycoproteins by stimulating the downstream FAK and ERK signaling molecules as mentioned above. In addition, the presence of OPN was also reported to enhance the collagen synthesis, cellular adhesion, proliferation, differentiation, angiogenesis, and calcification. Therefore, increased levels of OPN meant improved physiological processes and functions such as osteogenesis and angiogenesis. Both ALP and OPN were known as early markers of osteogenesis which was also clearly observed from our results. On the other hand, CS1 had significantly higher levels of OC only after 7 days of culture with approximately 1.9 and 1.5 times higher expressions when compared to CS0 and CS0.5, respectively (Figure 9C). OC is known to be the most abundant osteogenic specific non-collagenous protein in the extracellular matrix of bones. It is a late-stage osteogenic marker as it has a high affinity for calcium which plays an important role in mineralization. In addition, it was reported that OC also could function as a cell-signaling molecule in recruiting the osteoclasts and osteoblasts for bone resorption and remodeling. Our recent studies indicated that ALP, OPN, and OC could play a role as structural molecules in enhancing bone regeneration. Thus, further studies were required to observe the effects of CS and biomechanical stimulation on in vivo bone regeneration.

### 3.8. Cyclic Tensile Stimulated Osteogenic Differentiation of hPDLs through YAP

**Figure 10:**
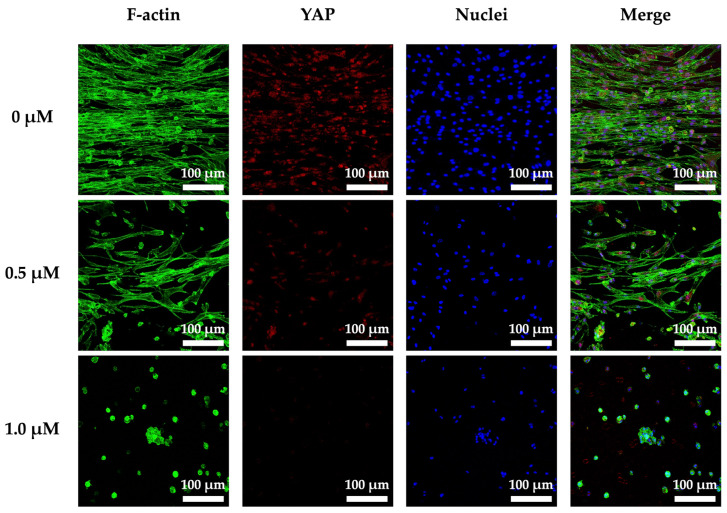
Immunofluorescence staining of YAP expression of hPDLs-laden CS1 auxetic scaffolds with cyclic tensile stimulation and YAP inhibitor (Veteprofin: 0, 0.5, and 1.0 µM) for 3 days. The scale bar is 100 μm.

As mentioned elsewhere in this study, cells are constantly exposed to both intrinsic and extrinsic stimuli which are integral to morphogenetic processes in embryological tissue development and regeneration [50,51,52]. In our study, to further explore the effect of cyclic tensile stimulation on osteogenic differentiation, we analyzed the expression of YAP protein and further understood the relevant mechanisms by using protein inhibitors (Figure 10). We quantified YAP activity by estimating its subcellular localization by immunofluorescence of nuclear and cytoplasmic signals. We found when a cyclic tension stimulus was loaded on the auxetic scaffold, the YAP of the cells was abundantly expressed as expected. In addition, the 0 µM groups had the highest expressions of YAP when compared to the 0.5 and 1 µM groups. In addition, YAP expressions were synchronous with the concentration of Verteporfin, and 1 µM had the lowest expressions of YAP and the least cell counts and aligned cells when compared to the rest of the groups. This result clearly indicated that the mechanical stimulation was able to regulate the cell behaviors capabilities via YAP expressions in the hPDLs-laden CS/FGelMa auxetic scaffold. An environment with disturbances in stimuli, such as modifications in extracellular matrix stiffness, could lead to pathological development of organs, by contributing to ageing and malignancy [53,54]. A study reported by Dupont et al. showed that the cells expressed higher levels of YAP when cultured on softer substrates when compared to the cells cultured on hard substrates, indicating that YAP expressions were regulated and influenced by the stiffness of extracellular matrix [55]. Their studies further proved that YAP were regulated by cell geometries and cytoskeletal stiffness and that expressions of YAP were critical for proper tissue regeneration.

**Figure 11:**
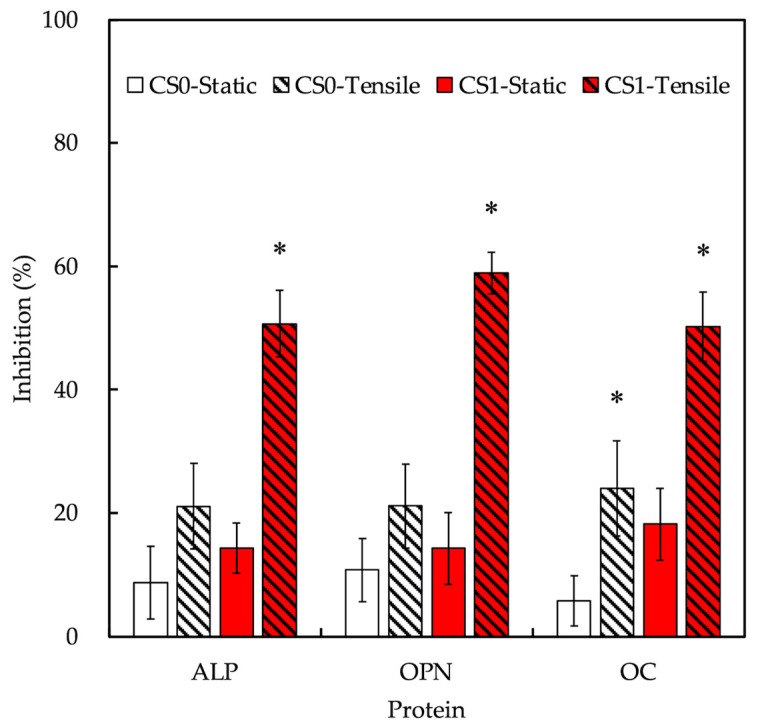
YAP inhibitors regulate the expression of three osteogenic-related proteins (ALP, OPN, and OC) in hPDLs-laden auxetic scaffolds with and without tension stimulation for 3 days. Data presented as mean ± SEM, *n* = 6 for each group. A *p*-value of <0.05 was considered as a significant difference according to the calculation of Scheffe’s multiple comparison test. * represents significant differences when compared to static.

The inhibition levels of osteogenic-related markers were evaluated after YAP inhibition and the results are shown in Figure 11. As seen, CS1-tensile had the highest and most significant levels of inhibition when compared to CS1-static. Inhibition levels of ALP, OPN, and OC for CS1-tensile were 50.7 ± 5.3%, 59.0 ± 3.4%, and 50.2 ± 5.6%, respectively, when compared to CS0-tensile of 21.1 ± 6.9%, 21.1 ± 6.8%, and 24.0 ± 7.7%. These data confirmed that the application of tensile stimulation increased expressions of osteogenic-related markers when compared to static cultures, which was similar as discussed above [56]. Secondly, the results also confirmed that the presence of CS improved the osteogenic capabilities as seen from the inhibition differences between CS0 and CS1. Most importantly, the results showed that the mechanical stimulation would work via YAP stimulation to increase the osteogenic capabilities.

## 4. Conclusions

In this study, we fabricated a CS/FGelMa auxetic scaffold using 3D printing and evaluated its osteogenic capabilities. Firstly, FTIR and XRD results showed that the CS could be incorporated into the FGelMa hydrogels by varying the concentrations of CS without affecting the structural integrity of FGelMa. Furthermore, the majority of the bonds between CS and FGelMa were covalent bonds, which were also considered responsible for improving the tensile strength and degradation rates of the CS/FGelMa hydrogels. The mechanical strength/elastic modulus of CS0, CS0.5, and CS1 were 20.7 ± 1.9/47.9 ± 3.5, 24.8 ± 1.6/64.0 ± 4.6, and 31.2 ± 2.1/75.1 ± 5.3 kPa, respectively. In addition, the degradation rates were decreased significantly with CS1 having 70.1 ± 1.3% of residual weight after 14 days of immersion, as compared to 59.6 ± 1.9% and 64.4 ± 2.1%, respectively, for CS0 and CS0.5. Furthermore, the presence of CS improved the initial adhesion and proliferation of hPDLs-laden auxetic scaffold via activating pFAK/FAK and pERK/ERK downstream factors for osteogenesis. The proliferation levels of CS1 were 45% and 16% higher than CS0 and CS0.5. Interestingly, our results further showed that the application of biomechanical stimulation to the auxetic scaffolds was able to further improve the adhesion and proliferation of hPDLs by at least 5% and was also able to enhance the secretion of osteogenic-related markers such as ALP, OPN, and OC. This prompted us to explore the related mechanism behind biomechanical stimulation. With the application of YAP staining and YAP inhibitors, we confirmed that the biomechanical stimulation could work via YAP receptor, which is a receptor for the mechanical cues and stimuli. Our results showed that the mechanical stimulation played a vital role in bone tissue regeneration and the novel scaffolds could be fabricated by combining the different aspects of science in order to bring the tissue engineering a step closer to the clinical applications.

## Data Availability

Data available in a publicly accessible repository.
